# Postoperative chemoradiotherapy versus chemotherapy for R0 resected gastric cancer with D2 lymph node dissection: an up-to-date meta-analysis

**DOI:** 10.1186/s12957-016-0957-7

**Published:** 2016-08-08

**Authors:** Meng-long Zhou, Mei Kang, Gui-chao Li, Xiao-mao Guo, Zhen Zhang

**Affiliations:** 1 Department of Radiation Oncology, Fudan University Shanghai Cancer Center, 270 Dong An Rd, Shanghai, 200032 PR China; 2 Department of Oncology, Shanghai Medical College, Fudan University, 270 Dong An Rd, Shanghai, 200032 PR China; 3 Institute of Clinical Epidemiology, Key Laboratory of Public Health Safety, Ministry of Education, School of Public Health, School of Public Health, Fudan University, 130 Dong An Rd, Shanghai, 200032 PR China

**Keywords:** Stomach neoplasms, Chemoradiotherapy, D2 surgery, Meta-analysis

## Abstract

**Background:**

This meta-analysis aims to provide more evidence on the role of postoperative chemoradiotherapy (CRT) for gastric cancer (GC) patients in Asian countries where D2 lymphadenectomy is prevalent.

**Methods:**

We conducted a systematic review of randomized controlled trials (RCTs), extracted data of survival and toxicities, and pooled data to evaluate the efficacy and toxicities of CRT compared with chemotherapy (CT) after D2 lymphadenectomy.

**Results:**

A total of 960 patients from four RCTs were selected. The results showed that postoperative CRT significantly reduced loco-regional recurrence rate (LRRR: RR = 0.50, 95 % CI = 0.34–0.74, *P* = 0.0005) and improved disease-free survival (DFS: HR = 0.73, 95 % CI = 0.60–0.89, *P* = 0.002). However, CRT did not affect distant metastasis rate (DMR: RR = 0.81, 95 % CI = 0.60–1.08, *P* = 0.15) and overall survival (OS: HR = 0.91, 95 % CI = 0.74–1.11, *P* = 0.34). The main grade 3–4 toxicities manifested no significant differences between the two groups.

**Conclusions:**

Overall, CRT after D2 lymphadenectomy may reduce LRRR and prolong DFS. The role of postoperative CRT should be further investigated in the population with high risk of loco-regional recurrence.

## Background

Despite its decline in the incidence over the past century, gastric cancer (GC) remains the second leading cause of cancer-related mortality worldwide and the most prevalent cancer in East Asia [1]. Surgical resection is considered the primary curative approach for this disease. However, even after radical resection, the loco-regional recurrence rate (LRRR) currently ranges from 24 % to 54 % [2], indicating that the effectiveness of surgery alone remains poor and unsatisfactory. Due to the high risk of loco-regional recurrence (LRR), issues about the extent of lymph node dissection and multimodality treatment have always been discussed.

During the past two decades, combined modality therapy has been widely investigated to prevent recurrence and improve survival for GC patients after curative resection. Due to the lack of powerful evidence regarding which of the current multimodality treatment is more beneficial, the standards of the postoperative and preoperative treatments differ around the world.

Perioperative chemotherapy (CT) (the MAGIC [3] and FFCD9703 [4] trials) and postoperative chemoradiotherapy (CRT) (the INT-0116 [5] trial) are recommended for resectable GC in Europe and North America, respectively. In Asian countries, postoperative CT is considered as the standard treatment based on the results of the ACTS-GC [6] and CLASSIC [7] trials.

D2 lymphadenectomy has been widely accepted in Asian countries, whereas two randomized controlled trials (RCTs) (the Dutch [8–10] and MRC [11, 12] trials) in the West did not demonstrate significant survival advantage of D2 lymphadenectomy over D1. Currently, D2 lymphadenectomy is recommended in Western countries if it is performed by well-trained surgeons with acceptable rates of postoperative mortality [13]. However, the INT0116 trial was initiated in 1991 and 90 % of patients received D0 or D1 lymphadenectomy in this trial. This was considered to be sub-optimal and thus, it cannot be arbitrarily used for reference in Asian countries, where D2 lymphadenectomy is widely performed.

After curative resection with D2 lymphadenectomy and postoperative CT, about 10 % of patients will still have LRR [6]. Therefore, it is meaningful to explore whether radiotherapy (RT) added to postoperative CT will further improve survival for GC patients after D2 curative resection. Only a few small prospective studies [14–16] and retrospective studies [17–22] have explored the role of postoperative CRT, but the results were inconsistent. Previously published meta-analyses [23–29] seldom included D2 lymph node dissection in their selection criteria. The team who conducted the ARTIST trial updated its follow-up results and for the first time reported the data of OS in their article published in 2015 [30]. Therefore, we conducted this meta-analysis based on the latest survival data, aiming to provide more evidence for this issue.

## Methods

### Literature search strategy

A systematic review of eligible RCTs was performed by searching the electronic databases, which consist of PubMed, EMBASE, Cochrane Library, and Web of Science. The keywords used for search were as follows: “gastric cancer,” “stomach neoplasms,” “chemoradiotherapy,” “combined modality therapy,” and “D2”. Search strategy was slightly adjusted according to the requirement of different databases. The search was limited to RCTs which were reported in English only. The deadline of this search was June 1, 2015. We also searched the annual meeting proceedings of ASCO, ESMO, and ASTRO. In addition, reference lists of systematic reviews and selected trials were scanned for any other possible relevant trials.

### Selection of trials

Two reviewers (Meng-long Zhou and Mei Kang) independently assessed every retrieved study for inclusion. All RCTs that compared CRT with CT in postoperative treatment for R0 resected GC with D2 lymphadenectomy were included in this meta-analysis. However, a preoperative CT or CRT is not allowed. When multiple publications by the same team from the same institution were found, the article that provided the most complete follow-up data on survival was selected.

### Quality assessment

Cochrane Collaboration’s tool was used for assessing risk of bias of RCTs (RoB tool, 5.1.0) [31]. RoB tool included the following index: sequence generation, allocation concealment, blinding of participants, personnel and outcome assessors, incomplete outcome data, selective outcome reporting, and other sources of bias. In all cases, an answer “Yes” indicated a low risk of bias, an answer “No” indicated high risk of bias, and if insufficient detail was reported of what happened in the study, the judgment would usually be “Unclear” risk of bias.

### Data extraction

The same two reviewers independently reviewed eligible studies for baseline characteristics and clinical relevance. Disagreements were resolved by an independent third reviewer (Gui-chao Li). The following variables were extracted from each trial onto standardized data collection forms if available: author, research title, year of publication, numbers of patients in each arm, baseline characteristics (age, sex, ECOG performance status, primary tumor site, Lauren classification, tumor stage), treatment (CT regimens, RT dose and technique, treatment completion), endpoints, length of follow-up, toxicities, and deaths.

### Statistical analysis

Survival variables were defined as generic inverse variance data. Hazard ratio (HR) and 95 % confidence interval (CI) for DFS and OS were extracted directly from the original article if possible. When HR and 95 % CI were not reported, they were calculated from published summary statistics or estimated from Kaplan-Meier survival curves using Tierney method [32]. Results regarding dichotomous data, such as loco-regional recurrence, distant metastasis, and toxicities, were reported as risk ratio (RR) with 95 % CI. The significance of the pooled data was determined by the *Z*-test, and a *P* value of less than 0.05 was considered as statistically significant.

Statistical heterogeneity between studies was examined using the chi-square-based *Q*-test and also expressed as *I*^2^. A *P* value of more than 0.10 for the *Q*-test and *I*^2^ of less than 50 % indicated a lack of heterogeneity across the trials. If there was no statistically significant heterogeneity in a given set of data, the fixed effects model was used. Otherwise, the random effects model was used. However, due to the fixed effects model tended to underestimate standard errors of pooled estimates, random effects model was used for the quantitative pooling [31, 33].

Publication bias was estimated by visually assessing the asymmetry of funnel plot. Furthermore, Egger’s test was also performed to provide quantitative evidence of publication bias [34]. A *P* value of less than 0.05 was considered representative of statistically significant publication bias. Sensitivity analysis was performed by sequentially omitting individual study to check the stability of the result. The statistical tests for our meta-analysis were performed with RevMan software (version 5.3, Cochrane).

## Results

### Trial flow and characteristics

**Fig. 1:**
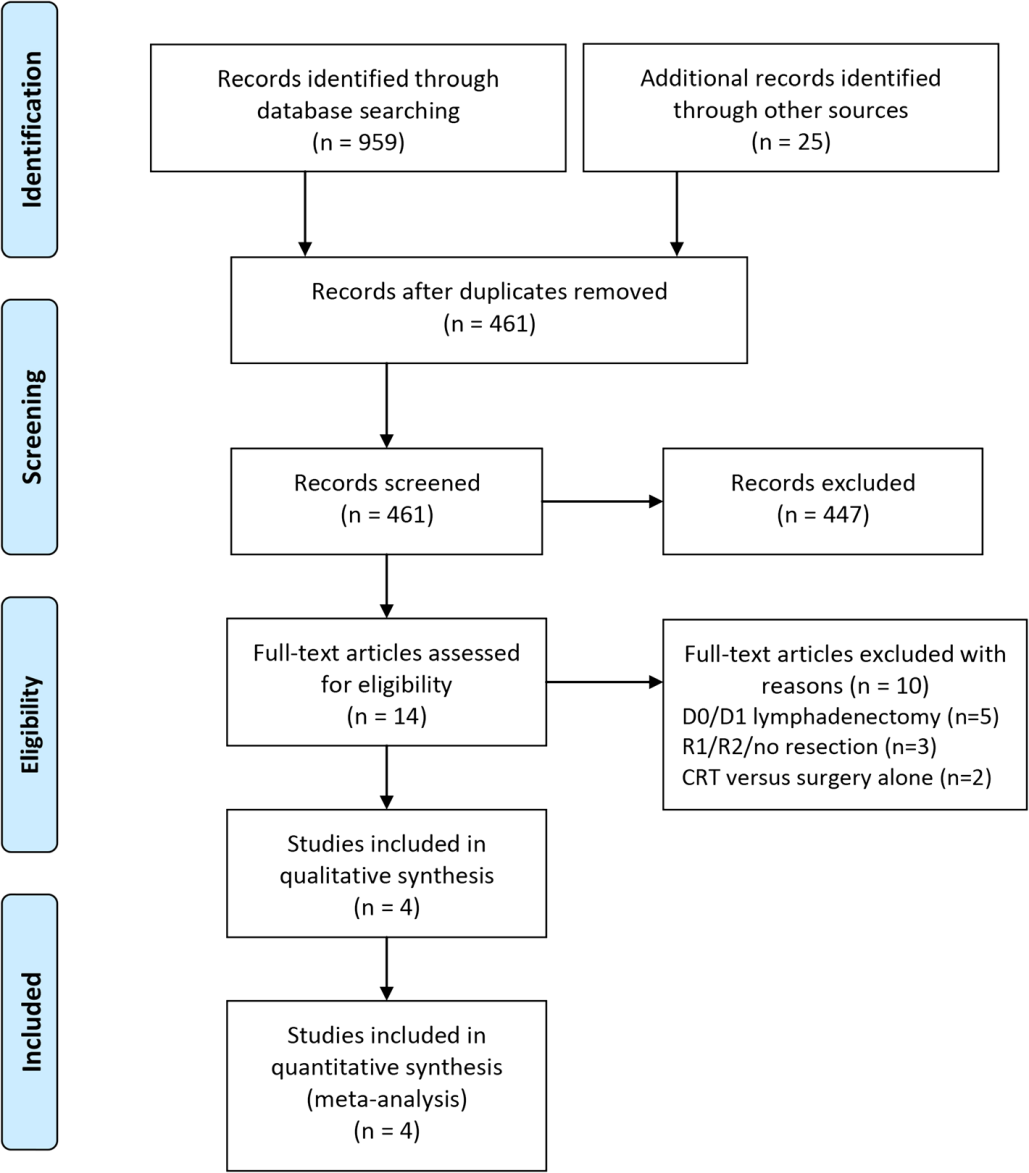
Flow chart of the study selection process

Finally, four RCTs met the inclusion criteria of this study. The included studies were published between 2010 and 2015 with the number of the enrolled patients ranging from 61 to 458. A total of 960 patients from four RCTs [14–16, 30] were identified for this meta-analysis at last. All studies were conducted in Asia (three [14, 15, 35] in Korea and one [16] in China). Two of them [15, 30] used AP-PA fields as a part of concurrent CRT, one [14] used three-dimensional conformal radiotherapy (3D-CRT) and one [16] applied intensity-modulated radiotherapy (IMRT). Two RCTs [14, 30] used cisplatin combined with capecitabine or fluorouracil as the CT regimens, and another two RCTs [15, 16] used the same CT regimen as that of the INT0116 trial. The PRISMA flow diagram of studies is shown in Fig. 1. Characteristics of the studies are summarized in Table 1.

**Table 1:** Characteristics of the included RCTs

References	Kwon et al. [14]		Kim et al. [15]		Zhu et al. [16]		ARTIST 2012/2015	
Characteristics	CRT	CT	CRT	CT	CRT	CT	CRT	CT
Patient number	31	30	46	44	186	165	230	228
Age year	56 (23–73)	49 (29–70)	>60, (19.6 %)	>60, (31.8 %)	56 (38–73)	59 (42–75)	56 (28–76)	56 (22–77)
Sex no. (%)								
Male	21 (67.7)	23 (76.7)	34 (73.9)	25 (56.8)	135 (72.9)	126 (76.4)	143 (62.2)	153 (67.1)
Female	10 (32.3)	7 (23.3)	12 (26.1)	19 (43.2)	51 (27.1)	39 (23.6)	87 (37.8)	75 (32.9)
ECOG PS no. (%)								
0	NR	NR	35 (76.1)	27 (61.4)	NR	NR	99 (43.0)	96 (42.1)
1	NR	NR	11 (23.9)	17 (38.6)	NR	NR	131 (57.0)	132 (57.9)
Primary tumor site no. (%)								
Proximal	6 (16.1)	4 (13.3)	3 (6.5)	2 (4.5)	30 (16.1)	15 (9.1)	13 (5.7)	9 (3.9)
Body	9 (29.0)	11 (36.7)	26 (56.5)	19 (43.2)	21 (11.3)	33 (20)	107 (46.5)	112 (49.1)
Antrum	17 (54.8)	15 (50.0)	14 (30.5)	18 (40.9)	135 (72.6)	117 (70.9)	90 (39.1)	87 (38.2)
Multiple/diffuse	0	0	3 (6.5)	5 (11.4)	0	0	20 (8.7)	20 (8.8)
Laurén’s classification no. (%)								
Intestinal	5 (16.1)	12 (40.0)	16 (34.8)	15 (34.1)	NR	NR	75 (32.6)	88 (38.6)
Diffuse	20 (64.4)	13 (43.3)	26 (56.5)	24 (54.5)	NR	NR	144 (62.6)	130 (57.0)
Mixed/unclassified	6 (19.4)	5 (16.7)	4 (8.6)	5 (11.3)	NR	NR	11 (4.8)	10 (4.4)
Tumor stage no. (%)								
Ib	0	0	0	0	20 (10.8)	15 (9.1)	49 (21.3)	50 (21.9)
II	0	0	0	0	36 (19.4)	30 (18.2)	84 (36.5)	86 (37.7)
III	24 (77.4)	27 (90.0)	34 (73.9)	31 (75.0)	103 (55.4)	96 (58.2)	71 (30.8)	65 (28.6)
IV (M0)	7 (22.6)	3 (10)	12 (26.1)	11 (25.0)	27 (14.5)	24 (14.5)	26 (11.3)	27 (11.8)
pN+	NR	NR	46 (100)	42 (95.5)	158 (84.9)	143 (86.7)	203 (88.2)	193 (84.6)
Lymphadenectomy	D2		D2		D2		D2	
Treatment regimens	FP/RT	FP	FL/RT	FL	FL/IMRT	FL	XP/XRT/XP	XP
Total RT dose/technique	45 Gy/3D-CRT		45 Gy/AP-PA fields		45 Gy/IMRT		45 Gy/AP-PA fields	
Endpoints	3-ys DFS: 80.0 % vs 75.2 %; *P* = 0.887 5-ys DFS: 76.7 % vs 59.1 %; *P* = 0.222 5-ys OS: 70.1 % vs 70.0 %; *P* = 0.814		5-ys DFS: 60.9 % vs 50.0 %; *P* = 0.246 5-ys OS: 65.2 % vs 54.6 %; *P* = 0.67		5-ys RFS: 45.2 % vs 35.8 %, *P* = 0.029 5-ys OS: 48.4 % vs 41.8 %, *P* = 0.122		3-ys DFS: 78.2 % vs 74.2 %; *P* = 0.0862 7-ys DFS: *P* = 0.740 5-ys OS: 75 % vs 73 %; *P* = 0.484	
Median follow-up months	77.2 (24–92.8)		86.7 (60.3–116.5)		42.5		2012: 53.2(36.9–77.3) 2015: 7 years	

FP regimen: 5-Fu 1000 mg/m^2^ continuous infusion on days 1–5, cisplatin 60 mg/m^2^ on day 1 every 3 weeks, totally 6 cycles; FP/RT: 1 cycle of FP, then RT (45 Gy of radiation at 1.8 Gy per day, 5 days per week, for 5 weeks with continuous capecitabine 825 mg/m^2^ twice daily during radiotherapy), followed by 3 cycles of FP; FL regimen: 5-Fu 425 mg/m^2^, leucovorin 20 mg/m^2^, for 5 days with a 4-week interval, totally 5 cycles; FL/RT: 1 cycle of FL, then RT (45 Gy of radiation at 1.8 Gy per day, 5 days per week, for 5 weeks with 2 cycles of FL), followed by 2 cycles of FL; XP regimen: capecitabine 1000 mg/m^2^ twice daily on days 1 to 14; cisplatin 60 mg/m^2^ on day 1 every 3 weeks, totally 6 cycles; XP/XRT/XP: 2 cycles of XP, then XRT (45 Gy of radiation at 1.8 Gy per day, 5 days per week, for 5 weeks with continuous capecitabine 825 mg/m^2^ twice daily during radiotherapy), followed by 2 cycles of XP

*CT* chemotherapy, *CRT* chemoradiotherapy, *NR* not reported, *3-ys DFS* 3-year disease-free survival, *5-ys DFS* 5-year disease-free survival, *5-ys OS* 5-year overall survival, *3D-CRT* 3D conformal radiotherapy, *IMRT* intensity-modulated radiotherapy, *LN* lymph node

### Quality assessment

**Fig. 2:**
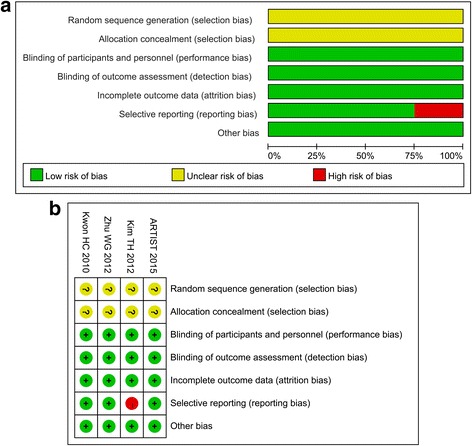
**a** Risk of bias graph: review authors’ judgments about each risk of bias item presented as percentages across all included studies. **b** Risk of bias summary: review authors’ judgments about each risk of bias item for each included study

Four RCTs were evaluated [14–16, 30, 35]: overall qualities were acceptable, and the baseline characteristics of patients were all reported. All RCTs mentioned “random", but none of them reported the details about random sequence generation which describes the method used to generate the allocation sequence in sufficient detail to allow an assessment of whether it should produce comparable groups. No RCT reported adequate allocation concealment which describes the method used to conceal the allocation sequence in sufficient detail to determine whether intervention allocations could have been foreseen in advance of, or during, enrolment. All RCTs were with low risks of incomplete outcome data. One RCT was with high risk of reporting bias due to its undetailed report of toxicities [15]. Blind method was not mentioned in all trials; however, this should unlikely affect the quality assessment results (Fig. 2).

### Patients’ survival data

**Fig. 3:**
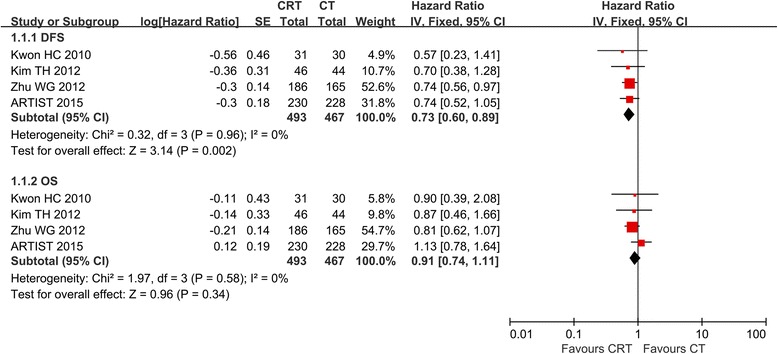
Forest plot of comparison: D2, R0 resection followed by postoperative chemoradiotherapy (*CRT*) versus D2, R0 resection followed by postoperative chemotherapy (*CT*). Outcomes: *1.1.1 DFS* is significantly improved. *1.1.2 OS* is not significantly improved

From four RCTs, 960 randomized patients, 493 in the CRT group and 467 in the CT group, were included in the meta-analyses of DFS and OS. The result of the test for heterogeneity of the treatment effects were non-significant (DFS: *P* = 0.96, *I*^2^ = 0 %; OS, *P* = 0.58, *I*^2^ = 0 %). Compared with CT, CRT significantly improved DFS (HR = 0.73, 95 % CI 0.60–0.89, *P* = 0.002). However, postoperative CRT did not have a significant positive effect on OS (HR = 0.91, 95 % CI 0.74–1.11, *P* = 0.34). The detailed data is shown in Fig. 3.

### Loco-regional recurrence and distant metastasis

**Fig. 4:**
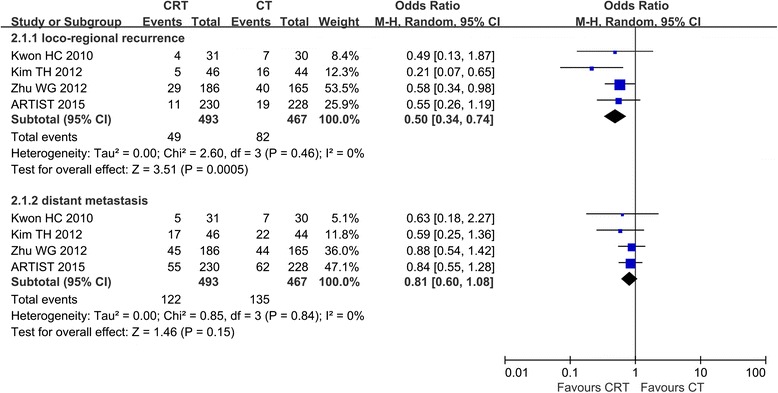
Forest plot of comparison: D2, R0 resection followed by postoperative chemoradiotherapy (*CRT*) versus D2, R0 resection followed by postoperative chemotherapy (*CT*). Outcomes: *2.1.1 Loco-regional recurrence* (LRR) is significantly decreased. *2.1.2 Distant metastasis* (DM) is not significantly decreased

Local-regional recurrence was reported in four studies, and the results of the meta-analysis indicated that there was a significant difference in LRRR between the two groups (RR = 0.50, 95 % CI 0.34–0.74, *P* = 0.0005) (Fig. 4). As for the distant metastasis rate (DMR), no significant difference was found between the two groups in our meta-analysis (RR = 0.81, 95 % CI 0.60–1.08, *P* = 0.15).

### Toxicities

Table 2 shows the main grade 3–4 toxicities occurred in each study. One trial [35] used common terminology criteria for adverse events (CTCAE version 2.0) to evaluate toxicities, another [16] used CTCAE version 3.0, while the other two trials [14, 15] did not mention the toxicity scale used. Differences among trials in the extent and methods of evaluation make statistical pooling of toxicities data impossible. Therefore, we only conducted a descriptive comparison from the available data. Generally, there were no significant differences in grade 3–4 toxicities between postoperative CRT and CT groups in three of the four trials [15, 16, 35]. However, Kwon et al. reported a higher rate of grades 3 and 4 neutropenia (48.4 %) with postoperative CRT than CT (16.7 %) [14].

**Table 2:** Main grade 3–4 toxicities of the included RCTs

References	Kwon et al. [14]		Kim et al. [15]		Zhu et al. [16]		ARTIST 2012/2015	
Characteristics	CRT	CT	CRT	CT	CRT	CT	CRT	CT
Patient number	31	30	46	44	186	165	230	228
Nausea/vomiting	2 (6.5)	4 (13.3)	NR	NR	8 (4.3)	0	35 (15.4)	36 (15.9)
Diarrhea	1 (3.2)	0	NR	NR	3 (1.6)	0	2 (0.9)	5 (2.2)
Neutropenia	15 (48.4)	5 (16.7)	NR	NR	14 (7.5)	12 (7.3)	110 (48.4)	92 (40.7)
Anemia	4 (12.9)	5 (16.7)	NR	NR	0	0	1 (0.4)	4 (1.7)
Thrombocytopenia	NR	NR	NR	NR	0	0	2 (0.9)	0
Criteria	NR		NR		CTCEA 3.0		CTCEA 2.0	

Numbers in the brackets refer to percentages

*CRT* chemoradiotherapy, *CT* chemotherapy, *NR* not reported

### Publication bias assessment

According to Cochrane Handbook for Systematic Reviews of Interventions [31], tests for funnel plot asymmetry should be used only when there are at least ten studies included in the meta-analysis; because when there are fewer studies, the power of the tests is too low to distinguish chance from real asymmetry. Therefore, the funnel plot was not given in this meta-analysis which included only four RCTs.

### Sensitivity analyses

Sensitivity analyses were performed to evaluate whether the pooled estimates of DFS, OS, LRRR, and DMR were different by exclusion of the highest weighted study and by omitting the RCTs that only included stage III/IV GC in each pooled analysis. Finally, the results were stable and all consistent with the above outcomes.

## Discussion

### Main findings

GC is a heterogeneous malignancy with poor prognosis and diverse treatment patterns globally [36]. Surgery is accepted as the primary treatment but the outcome remains dismal [37]. In Asian countries, several RCTs [7, 38] and meta-analyses [39, 40] have proven the efficacy of postoperative CT. In North America, the INT-0116 trial demonstrated the efficacy of postoperative CRT compared with surgery alone for the treatment of resectable GC. An observational study from Korea implies that postoperative CRT can prolong survival and decrease recurrence compared with observation after D2 surgery [18]. Additionally, several retrospective studies [20–22, 41] about postoperative CRT have been reported. However, all the abovementioned studies cannot settle the issue raised in our meta-analysis, that is, will postoperative CRT further improve survival compared with postoperative CT after R0 gastrectomy with D2 lymphadenectomy. Relevant RCTs [14–16, 30] are scarce, and controversy still exists. The ARTIST trial, which was designed to settle this issue, yielded negative results. However, given the default limitations in its trial design, the role of postoperative CRT after D2 dissection remains undefined. Thus, we performed an up-to-date meta-analysis, which summarized all the valuable data of the existing RCTs, aiming to provide more powerful results directing standard treatment for GC.

The present study showed that postoperative CRT, compared with postoperative CT, can improve LRRR and DFS, but did not improve DMR and OS. The benefit brought by CRT has not transformed into a better OS and a series of plausible reasons for this interesting finding are as follows: first, distant metastasis (DM) is the predominant recurrence pattern after D2 lymphadenectomy in the Asian population [42]. In contrast, LRR was more frequent than DM in the Western population who underwent D2 gastrectomy [43]. Second, D2 lymphadenectomy produces more reduction of LRR than that of DM, which can be supported by the results of the Dutch Gastric Cancer Group Trial [9] and the Taiwanese trial [44]. Moreover, postoperative CRT, as a local treatment alike to D2 lymphadenectomy, does not reduce DM even compared with observation [5, 18, 45]. Therefore, Huang et al. [23] maintained that the DM might offset the loco-regional control brought by CRT for Asian patients underwent D2 gastrectomy. Indeed, we can observe in these four RCTS that the DMR is higher than LRRR (16.1–37.0 vs. 4.8–15.6 %). Huang et al. considered that a high percentage of diffuse-type GC, which is prone to early metastasis, is the reasons why DM is the predominant recurrence pattern in the Asian population [46]. Around 60 % of patients in three of the four RCTs included in our meta-analysis were diffuse-type GC. This is also parallel with the result of a meta-analysis which reported that there is a significantly higher percentage of diffuse-type GC in the Asian population, which accounted for more than 50 % [47]. However, this explanation is inconsistent with the results of a RCT which proved that postoperative RT was effective to patients with diffuse-type GC [48]. Therefore, this controversy, which is raised by the INT-0116 trial decades ago, is still not well settled up to now.

In the ARTIST trial, patients with stage Ib and II GC accounted for nearly 60 % in both arms. Fan et al. considered that patients in relatively early stages with a lower risk of LRR in the study may dilute the survival benefit of CRT [49]. However, Dai et al. gave an opposite explanation of why improved loco-regional control has not transferred to OS benefit. They considered that patients with stages III–IV (M0) took a big proportion which is no less than 60 %, and the prognosis was still poor for patients at an advanced stage even if LRR was controlled since the lymph node metastasis occurred more frequently and the nutritional status was worse [28]. In addition to TNM stage, status of lymph nodes is associated with the efficacy of CRT. According to the subgroup analysis of the ARTIST trial, patients with positive lymph nodes could benefit from postoperative CRT. A retrospective study by Costa et al. also came to the same conclusion [21]. Inconsistently, although almost all patients in the trial conducted by Kim et al. [18] had positive lymph nodes (pN+, 100 % in the CRT group and 95.5 % in the CT group), it did not produce significantly improved DFS and OS. This may possibly ascribe to the small sample size, which was smaller than the estimated one and decreased the likelihood of finding a significant difference between the two groups. However, this is only our assumption, and we hope the ongoing ARTIST II trial could answer this question [50].

At present, we should not arbitrarily negate the effect of CRT after D2 dissection. It may be important and worthwhile to select patients at high risk of recurrence who would benefit from postoperative CRT. The ARTIST II trial [50] and another trial [51] conducted in China may better define the role of postoperative CRT in GC treated with D2 lymphadenectomy as they both focus on patients with stages II–III (according to the 7th AJCC staging system) disease and positive lymph nodes (pN+), and limit the surgery type to D2 lymph node dissection. These two trials are still enrolling patients, and we are looking forward to their preliminary results. Yu et al. [52] analyzed the results of the ARTIST trial, precisely defined the recurrent sites and found that postoperative CRT after D2 resection in GC reduced regional recurrence, especially in group 3 lymph nodes. However, no difference was observed between the two arms regarding the local recurrence, which may ascribe to that the remnant stomach was not considered a routine RT target in the present study. Therefore, for all the patients with positive lymph nodes, they can also be further stratified by the sites of involved lymph nodes and more prospective studies are required to evaluate the optimal RT target.

### Limitations

First, only four eligible trials were selected and this possibly could not unveil the real situation. Second, baseline characteristics of patients were similar among selected trials, except for tumor stage. Patients with stages III and IV (M0) were included in two selected trials (Kwon et al. [14] and Kim et al. [15] trials), but stage Ib–IV GC was included in Zhu et al. [16] and the ARTIST trial. However, sensitivity analyses showed that the results of meta-analysis are stable. Third, the RT techniques applied were different, including conventional AP-PA fields, 3D-CRT, and IMRT. Additionally, CT regimens differed. Compared with the present commonly used XELOX (oxaliplatin plus capectabine) or SOX (oxaliplatin plus S-1) regimens, the cytotoxic drugs used in the included RCTs may have relatively low efficacy with high toxicities. Fourth, the Kwon et al. [14] trial has high risk of reporting bias. Generally, regarding these limitations mentioned above, results should be cautiously interpreted.

## Conclusions

In sum, postoperative CRT after D2 surgery may benefit loco-regional control and improve DFS. However, it cannot bring survival benefit in an unselected group of patients. The role of postoperative CRT should be further investigated in the population with high risk of LRR.

## Abbreviations

3D-CRT, three-dimensional conformal radiotherapy; CI, confidence interval; CRT, chemoradiotherapy; CT, chemotherapy; DFS, disease-free survival; DM, distant metastasis; DMR, distant metastasis rate; GC, gastric cancer; HR, hazard ratio; IMRT, intensity-modulated radiotherapy; LRR, loco-regional recurrence; LRRR, loco-regional recurrence rate; OS, overall survival; RCT, randomized controlled trial; RR, risk ratio; RT, radiotherapy

## References

[1] Karimi P, Islami F, Anandasabapathy S, Freedman ND, Kamangar F (2014). Gastric cancer: descriptive epidemiology, risk factors, screening, and prevention. Cancer Epidemiol Biomarkers Prev.

[2] Lowy AM, Feig BW, Janjan N (2001). A pilot study of preoperative chemoradiotherapy for resectable gastric cancer. Ann Surg Oncol.

[3] Cunningham D, Allum WH, Stenning SP (2006). Perioperative chemotherapy versus surgery alone for resectable gastroesophageal cancer. N Engl J Med.

[4] Ychou M, Boige V, Pignon JP (2011). Perioperative chemotherapy compared with surgery alone for resectable gastroesophageal adenocarcinoma: an FNCLCC and FFCD multicenter phase III trial. J Clin Oncol.

[5] Macdonald JS, Smalley SR, Benedetti J (2001). Chemoradiotherapy after surgery compared with surgery alone for adenocarcinoma of the stomach or gastroesophageal junction. New Engl J Med.

[6] Sakuramoto S, Sasako M, Yamaguchi T (2007). Adjuvant chemotherapy for gastric cancer with S-1, an oral fluoropyrimidine. N Engl J Med.

[7] Bang YJ, Kim YW, Yang HK (2012). Adjuvant capecitabine and oxaliplatin for gastric cancer after D2 gastrectomy (CLASSIC): a phase 3 open-label, randomized controlled trial. Lancet.

[8] Songun I, Putter H, Kranenbarg EM, Sasako M, van de Velde CJ (2010). Surgical treatment of gastric cancer: 15-year follow-up results of the randomized nationwide Dutch D1D2 trial. Lancet Oncol.

[9] Hartgrink HH, van de Velde CJ, Putter H (2004). Extended lymph node dissection for gastric cancer: who may benefit? Final results of the randomized Dutch gastric cancer group trial. J Clin Oncol.

[10] Bonenkamp JJ, Hermans J, Sasako M (1999). Extended lymph-node dissection for gastric cancer. N Engl J Med.

[11] Cuschieri A, Weeden S, Fielding J (1999). Patient survival after D1 and D2 resections for gastric cancer: long-term results of the MRC randomized surgical trial. Surgical Co-operative Group. Br J Cancer.

[12] Cuschieri A, Fayers P, Fielding J (1996). Postoperative morbidity and mortality after D1 and D2 resections for gastric cancer: preliminary results of the MRC randomized controlled surgical trial. The Surgical Cooperative Group. Lancet.

[13] Chang JS, Koom WS, Lee Y, Yoon HI, Lee HS (2014). Postoperative adjuvant chemoradiotherapy in D2-dissected gastric cancer: is radiotherapy necessary after D2-dissection?. World J Gastroenterol.

[14] Kwon HC, Kim MC, Kim KH (2010). Adjuvant chemoradiation versus chemotherapy in completely resected advanced gastric cancer with D2 nodal dissection. Asia Pac J Clin Oncol.

[15] Kim TH, Park SR, Ryu KW (2012). Phase 3 trial of postoperative chemotherapy alone versus chemoradiation therapy in stage III-IV gastric cancer treated with R0 gastrectomy and D2 lymph node dissection. Int J Radiat Oncol Biol Phys.

[16] Zhu WG, Xua DF, Pu J (2012). A randomized, controlled, multicenter study comparing intensity-modulated radiotherapy plus concurrent chemotherapy with chemotherapy alone in gastric cancer patients with D2 resection. Radiother Oncol.

[17] Vaishampayan UN, Ben-Josef E, Philip PA (2002). A single-institution experience with concurrent capecitabine and radiation therapy in gastrointestinal malignancies. Int J Radiat Oncol Biol Phys.

[18] Kim S, Lim DH, Lee J (2005). An observational study suggesting clinical benefit for adjuvant postoperative chemoradiation in a population of over 500 cases after gastric resection with D2 nodal dissection for adenocarcinoma of the stomach. Int J Radiat Oncol Biol Phys.

[19] Tsang WK, Leung SF, Chiu SK (2007). Adjuvant chemoradiation for gastric cancer: experience in the Chinese population. Clin Oncol (R Coll Radiol).

[20] Leong CN, Chung HT, Lee KM (2008). Outcomes of adjuvant chemoradiotherapy after a radical gastrectomy and a D2 node dissection for gastric adenocarcinoma. Cancer J.

[21] Costa WJ, Coimbra FJ, Fogaroli RC, et al. Adjuvant chemoradiotherapy after d2-lymphadenectomy for gastric cancer: the role of n-ratio in patient selection. Results of a single cancer center. Radiat Oncol. 2012;7:169 10.1186/1748-717X-7-169 PMC3542168 23068190

[22] Jacome AA, Wohnrath DR, Scapulatempo NC (2013). Effect of adjuvant chemoradiotherapy on overall survival of gastric cancer patients submitted to D2 lymphadenectomy. Gastric Cancer.

[23] Huang Y, Yang Q, Zhou S (2013). Postoperative chemoradiotherapy versus postoperative chemotherapy for completely resected gastric cancer with D2 lymphadenectomy: a meta-analysis. Plos One.

[24] Ronellenfitsch U, Schwarzbach M, Hofheinz R (2013). Preoperative chemo(radio)therapy versus primary surgery for gastroesophageal adenocarcinoma: systematic review with meta-analysis combining individual patient and aggregate data. Eur J Cancer.

[25] Ohri N, Garg MK, Aparo S (2013). Who benefits from adjuvant radiation therapy for gastric cancer? A meta-analysis. Int J Radiat Oncol Biol Phys.

[26] Yang Q, Wei Y, Chen Y, Zhou S, Jiang Z, Xie D. Indirect comparison showed survival benefit from adjuvant chemoradiotherapy in completely resected gastric cancer with D2 lymphadenectomy. Gastroent Res Pract. 2013;2013:1-7 10.1155/2013/634929 PMC3806404 24194750

[27] Liang JW, Zheng ZC, Yu T, Wang X, Zhang JJ (2014). Is postoperative adjuvant chemoradiotherapy efficacious and safe for gastric cancer patients with D2 lymphadenectomy? A meta-analysis of the literature. Eur J Surg Oncol.

[28] Dai Q, Jiang L, Lin R (2015). Adjuvant chemoradiotherapy versus chemotherapy for gastric cancer: a meta-analysis of randomized controlled trials. J Surg Oncol.

[29] Soon YY, Leong CN, Tey JCS, Tham IWK, Lu JJ. Postoperative chemo-radiotherapy versus chemotherapy for resected gastric cancer: a systematic review and meta-analysis. J Med Imag Radiat On. 2014; 4: n/a-n/a. 10.1111/1754-9485.12190 24995607

[30] Park SH, Sohn TS, Lee J, et al. Phase III Trial to compare adjuvant chemotherapy with capecitabine and cisplatin versus concurrent chemoradiotherapy in gastric cancer: final report of the adjuvant chemoradiotherapy in stomach tumors trial, including survival and subset analyses. J Clin Oncol. 2015;33(28):3130-3136. 10.1200/JCO.2014.58.3930 25559811

[31] The Cochrane Collaboration: Handbook for systematic reviews of interventions Version 5.1.0. 2011.

[32] Tierney JF, Stewart LA, Ghersi D, Burdett S, Sydes MR. Practical methods for incorporating summary time-to-event data into meta-analysis. Trials. 2007;8:16. 10.1186/1745-6215-8-16 PMC1920534 17555582

[33] Lau J, Ioannidis JP, Schmid CH (1997). Quantitative synthesis in systematic reviews. Ann Intern Med.

[34] Egger M, Davey SG, Schneider M, Minder C (1997). Bias in meta-analysis detected by a simple, graphical test. BMJ.

[35] Lee J, Lim DH, Kim S (2012). Phase III trial comparing capecitabine plus cisplatin versus capecitabine plus cisplatin with concurrent capecitabine radiotherapy in completely resected gastric cancer with D2 lymph node dissection: the ARTIST trial. J Clin Oncol.

[36] Shah MA, Ajani JA (2010). Gastric cancer—an enigmatic and heterogeneous disease. JAMA.

[37] Roukos DH, Kappas AM (2005). Perspectives in the treatment of gastric cancer. Nat Clin Pract Oncol.

[38] Sasako M, Sakuramoto S, Katai H (2011). Five-year outcomes of a randomized phase III trial comparing adjuvant chemotherapy with S-1 versus surgery alone in stage II or III gastric cancer. J Clin Oncol.

[39] Paoletti X, Oba K, Burzykowski T (2010). Benefit of adjuvant chemotherapy for resectable gastric cancer. JAMA.

[40] Wagner AD (2006). Chemotherapy in advanced gastric cancer: a systematic review and meta-analysis based on aggregate data. J Clin Oncol.

[41] Ejaz A, Spolverato G, Kim Y (2014). Impact of external-beam radiation therapy on outcomes among patients with resected gastric cancer: a multi-institutional analysis. Ann Surg Oncol.

[42] Tsujinaka T, Fujitani K, Hirao M, Kurokawa Y (2008). Current status of chemoradiotherapy for gastric cancer in Japan. Int J Clin Oncol.

[43] Cirera L, Balil A, Batiste-Alentorn E (1999). Randomized clinical trial of adjuvant mitomycin plus tegafur in patients with resected stage III gastric cancer. J Clin Oncol.

[44] Wu CW, Hsiung CA, Lo SS (2006). Nodal dissection for patients with gastric cancer: a randomized controlled trial. Lancet Oncol.

[45] Smalley SR, Benedetti JK, Haller DG (2012). Updated analysis of SWOG-directed intergroup study 0116: a phase III trial of adjuvant radiochemotherapy versus observation after curative gastric cancer resection. J Clin Oncol.

[46] Brooks GA, Enzinger PC, Fuchs CS (2012). Adjuvant therapy for gastric cancer: revisiting the past to clarify the future. J Clin Oncol.

[47] Hsu C, Shen YC, Cheng CC, Cheng AL, Hu FC, Yeh KH (2012). Geographic difference in safety and efficacy of systemicchemotherapy for advanced gastric or gastroesophagealcarcinoma: a meta-analysis and meta-regression. Gastric Cancer.

[48] Stessin AM, Sison C, Schwartz A, Ng J, Chao CK, Li B (2014). Does adjuvant radiotherapy benefit patients with diffuse-type gastric cancer? Results from the surveillance, epidemiology, and end results database. Cancer-Am Cancer Soc.

[49] Fan M, Hu W, Zhang Z. Chemoradiation for gastric cancer: controversies, updates and novel techniques. Br J Radiol. 2015;88(1051):20150027. 10.1259/bjr.20150027 PMC4628527 25827208

[50] Phase III randomized trial of adjuvant chemotherapy with S-1 vs S-1/oxaliplatin +/− radiotherapy for completely resected gastric adenocarcinoma: The ARTIST II Trial. J Clin Oncol. 2015;suppl3. abstr TPS228.

[51] Phase III randomized trial of adjuvant XELOX chemotherapy and XELOX with concurrent capecitabine and radiotherapy for gastric adenocarcinoma with D2 dissection. 2012. Available from: http://clinicaltrials.gov/ct2/show/NCT01711242.

[52] Yu JI, Lim DH, Ahn YC, et al. Effects of adjuvant radiotherapy on completely resected gastric cancer: a radiation oncologist’s view of the ARTIST randomized phase III trial. Radiother Oncol. 2015;117(1):171–77. 10.1016/j.radonc.2015.08.009 26299196

